# Impact of perioperative administration of 6 % hydroxyethyl starch 130/0.4 on serum cystatin C-derived renal function after radical prostatectomy: a single-centre retrospective study

**DOI:** 10.1186/s12871-016-0236-8

**Published:** 2016-08-30

**Authors:** Stefan Südfeld, Sami R. Leyh-Bannurah, Lars Budäus, Markus Graefen, Philip C. Reese, Franziska von Breunig, Daniel A. Reuter, Bernd Saugel

**Affiliations:** 1 Department of Anesthesiology, Center of Anesthesiology and Intensive Care Medicine, University Medical Center Hamburg-Eppendorf, Martinistrasse 52, 20246 Hamburg, Germany; 2 Martini Clinic, Prostate Cancer Center at University Medical Center Hamburg-Eppendorf, Martinistrasse 52, 20246 Hamburg, Germany

**Keywords:** Acute kidney injury, Anesthesia, Kidney function tests, Perioperative period

## Abstract

**Background:**

Hydroxyethyl starch (HES) is used for repletion of acute intravasal volume loss in surgical patients. However, in critically ill patients, HES is associated with acute kidney injury. We aimed to evaluate the effect of HES on perioperative cystatin C (cystC)-derived estimated glomerular filtration rates (eGFR_cystC_) in patients undergoing open and robot-assisted radical prostatectomy.

**Methods:**

In this retrospective study we included 179 patients who underwent general anaesthesia for radical prostatectomy received HES perioperatively, and had complete cystC and fluid therapy data available. CystC and corresponding eGFR_cystC_ at postoperative days 1, 3, and 5 were compared with preoperative baseline using Wilcox rank-sum test.

**Results:**

In 179 eligible patients, 6 % HES 130/0.4 was administered in a median (25th to 75th percentile) dose of 1000 mL (1000 to 1000 mL). Baseline eGFR_cystC_ was 109.4 mL/min (100.3 to 118.7 mL/min). eGFR_cystC_ on postoperative days 1, 3, and 5 was 120.4 mL/min (109.4 to 134.0 mL/min), 120.4 mL/min (109.4 to 132.9 mL/min), and 117.9 mL/min (106.6 to 129.8 mL/min), respectively (*p* < 0.001 compared with baseline, each). No patient had an eGFR_cystC_-decrease of ≥25 % from baseline.

**Conclusions:**

The results indicate that the administration of a median dose of 1000 mL of 6 % HES 130/0.4 is not associated with a postoperative deterioration of renal function in patients with normal to near-normal baseline renal function undergoing radical prostatectomy.

## Background

Postoperative acute kidney injury (AKI) is highly prevalent [1] and associated with increased hospital mortality [2]. Multiple factors contribute to AKI, e.g. the type of surgery and haemodynamic instability [3]. Moreover, recent studies suggested that the administration of fluids containing hydroxyethyl starch (HES) leads to a moderately increased risk of AKI and increased requirement of renal replacement therapy in critically ill patients [4–6]. In the operative setting, on the contrary, this connection is currently not clearly evident [7]. In patients undergoing major surgery, prospective studies did not show a considerable effect of perioperatively administered HES on estimated glomerular filtration rate (eGFR) calculated based on serum creatinine (sCr) [8–10].

Similar results were obtained when using the more sensitive [11, 12] glomerular filtration rate (GFR)-marker cystatin C (cystC) [13, 14]. Therefore, a possible context-dependency (i.e. elective surgery vs. critical illness) of the degree of apparent adverse effects of HES on kidney function may exist.

We hypothesised that perioperative administration of HES for the replacement of acute intravasal volume loss due to intraoperative haemorrhage does not lead to clinically relevant impairment of eGFR in a population of elective surgical patients without marked a priori risk of AKI. This in itself would be an important finding for a more detailed understanding of differential indications and contraindications of HES.

Therefore, we evaluated the influence of perioperatively administered HES on eGFR determined by sequential cystC measurements (eGFR_cystC_) in a general patient population undergoing open radical prostatectomy (ORP) or robot-assisted radical prostatectomy (RARP) in a specialised prostate cancer centre at a large university hospital.

## Methods

### Study design

The study protocol of this retrospective analysis (ethics committee number PV4998) was reviewed and approved by the appropriate ethics committee (Ethikkommission der Ärztekammer Hamburg, Hamburg, Germany). Due to the retrospective nature of the study and the anonymisation of data the need for informed consent was waived by the ethics committee.

To evaluate the influence of perioperatively administered HES on eGFR_cystC_ in patients undergoing ORP or RARP, we retrospectively extracted and analysed data from the hospital-wide digital patient record system that included biometric, medical, procedural, and physiologic parameters of patients in whom perioperative cystC measurements had been performed during their hospitalisation for ORP or RARP between September 2012 and April 2013.

### Patients; inclusion/exclusion criteria

Patients were eligible for study inclusion if they a) had undergone general anaesthesia for ORP or RARP (without the need for a re-intervention within 5 days from the original operation), b) had received HES perioperatively, c) had cystC measurements recorded in their electronic hospital charts (at least one cystC value measured preoperatively (baseline) and at least one cystC value measured on days 1 or 3), and d) had complete documentation of perioperative fluid therapy in the digital patient record system.

### Data acquisition

We extracted data on the type of radical prostatectomy (ORP vs. RARP), duration of surgery, intraperitoneal pressure (in RARP), and estimated intraoperative blood loss from surgical records. Biometric and medical status data, including relevant pre-existing co-morbidities and long-term medication, were obtained from preoperative anaesthesiological evaluation notes. For the operative period (including post anaesthesia care unit), we extracted data from the corresponding anaesthesia records. Laboratory data were extracted from the digital patient record system for preoperative baseline and days 1, 3, and 5. Baseline systolic arterial blood pressure (SAP) was defined as the first SAP measurement at the time of the patients’ arrival in the anaesthesia induction area. Doses of norepinephrine were expressed as the maximum infusion rate during the time under anaesthetic care. For perioperative fluid and volume therapy, the colloid 6 % HES 130/0.4 (Vololyte®; Fresenius Kabi Deutschland GmbH; Bad Homburg, Germany) and the balanced full electrolyte solution (Sterofundin®; B. Braun Melsungen AG, Melsungen, Germany) were used. The documented units of intravenous fluids (i.e. crystalloids, colloids, or blood products) were assumed to be administered completely without residue.

### Calculation of estimated glomerular filtration rate from serum cystatin C

For the calculation of eGFR_cystC_, we used the formula previously published by Le Bricon and colleagues: [15].

eGFR_cystC_ (mL/min) = 78 × cystC [mg/L]^−1^ + 4.

For eGFR derived from concomitant sCr measurements (eGFR_crea_), we applied the simplified Modification of Diet in Renal Disease (MDRD-4)-formula: [16].

eGFR_crea_ (mL/min) = 186 × sCr [mg/dL]^-1,154^ × age [yrs]^-0,203^, corrected for sex and race (× 1, if male Caucasian). If not stated in the patient records, the patient’s race was assumed to be Caucasian. All eGFR_x_-values stated in this publication are normalised for 1.73 m^2^ body surface area.

### Statistical analysis

We present descriptive statistical analyses as median (25th to 75th percentile range) for continuous data and as absolute frequencies with percentages for categorical data. Within-group differences were evaluated using Wilcox signed rank test for paired non-parametric data. Box-whisker-plots were created for illustration of chosen non-parametric data. For data management we used Microsoft Excel 2010 (Microsoft Corp., Redmond, WA, USA) and for statistical analyses and figures we used IBM SPSS Statistics, Version 21.0 (IBM Corp., Armonk, NY, USA).

Cases, in which data were missing, were excluded in the respective analysis. Statistical significance was assumed for *p* < 0.05.

## Results

### Patients

**Fig. 1:**
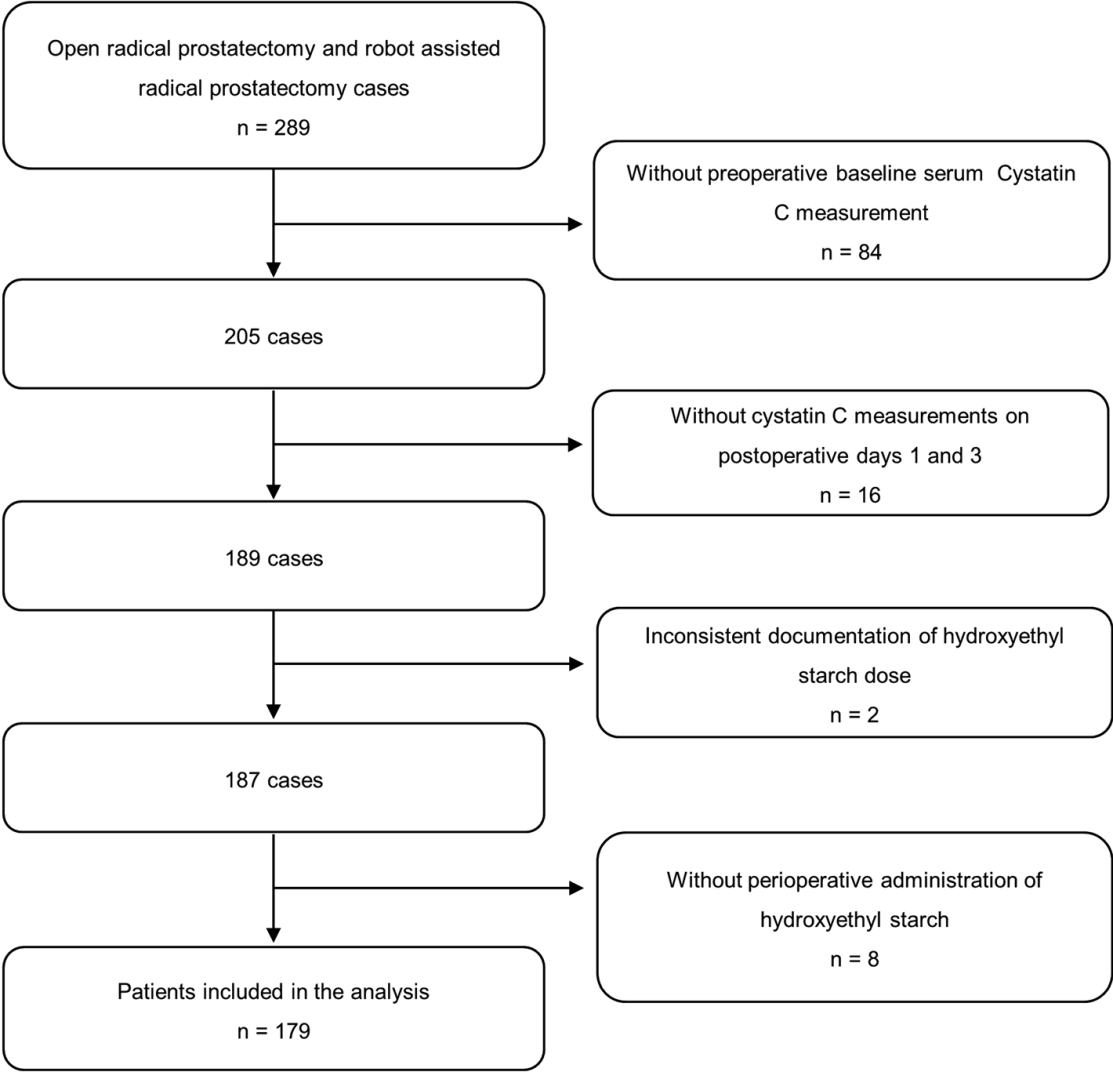
Patient flow diagram. Illustration of patient exclusion

In this study we analysed 179 patients (Fig. 1). Of note, 8 Patients were excluded a priori that did not receive HES perioperatively. We present the patients’ characteristics in Table 1.

**Table 1:** Patient characteristics

Parameter, unit	Value
Age, yrs	64 (59 to 69)
Weight, kg	83.0 (76.0 to 90.0)
Height, m	1.8 (1.75 to 1.83)
ASA physical status classification	
I	12 (6.7)
II	115 (64.2)
III	43 (24.0)
IV	1 (0.6)
V	0 (0.0)
VI	0 (0.0)
Medical co-morbidities	
Chronic arterial hypertension	80 (44.7)
Chronic heart failure	1 (0.6)
Coronary artery disease	7 (3.9)
Cerebrovascular disease	11 (6.1)
Periphery artery disease	1 (0.6)
Atrial fibrillation	6 (3.4)
Chronic kidney disease	1 (0.6)
Diabetes mellitus	14 (7.8)
Rheumatoid arthritis	2 (1.1)
Long-term Medication	
Diuretic (thiazide, indapamid)	17 (9.5)
Diuretic (K^+^-sparing)	2 (1.2)
Diuretic (loop of Henle)	0 (0.0)
ACE-inhibitor	26 (14.5)
AT1-blocker	41 (22.9)
Renin-antagonist	1 (0.6)
β-blocker	29 (16.2)
Calcium channel-blocker	22 (12.3)
α_1_-blocker	20 (11.2)
α_2_-agonist	3 (1.7)
NSAID	25 (13.9)

Data presented as median (25th to 75th percentile) or number of cases (%)

*ASA* American Society of Anesthesiologists, *ACE* angiotensin-converting-enzyme, *AT1* angiotensin-receptor-1, *NSAID* non-steroidal anti-inflammatory drug

### Procedural data

Procedural data of the study population, including haemodynamic therapy, are presented in Table 2.

**Table 2:** Procedural data

Parameter, unit	Value
Operation	
Robot-assisted radical prostatectomy	69 (38.5)
Open radical prostatectomy	110 (61.5)
Duration of surgery, min	190 (164 to 225)
Intraperitoneal pressure, if applicable, mmHg	15 (15 to 15)
Mode of anaesthesia	
Total intravenous	86 (48.0)
Balanced	93 (52.0)
Spinal (combined)	72 (40.2) (all ORP)
Haemodynamic data	
Blood loss (intraoperative), mL	500 (250 to 800)
Diuresis in PACU, mL	800 (550 to 1200)
Pre-induction SAP, mmHg	130 (120 to 140)
SAP ≤ 100 mmHg, n	174 (97.2)
SAP ≤ 90 mmHg, n	104 (58.1)
SAP ≤ 80 mmHg, n	38 (21.2)
SAP ≤ 70 mmHg, n	7 (3.9)
SAP ≤ 60 mmHg, n	1 (0.6)
Haemodynamic therapy	
HES solution, mL	1000 (1000 to 1000)
Crystalloid solution, mL	3500 (2500 to 3500)
Cases administered RCCs	4 (2.3)
Number of RCCs, if administered, n	2.0 (1.3 to 2.0)
Cases administered FFPs, n	0 (0.0)
Cases administered PCs, n	0 (0.0)
Maximum norepinephrine dose, μg/min	6.0 (5.0 to 9.0)

Data presented as median (25th to 75th percentile) or number of cases (%)

*ORP* open radical prostatectomy, *PACU* post anaesthesia care unit, *SBP* = systolic arterial blood pressure, *HES* hydroxyethyl starch, *RCC* red cell concentrate, *FFP* fresh frozen plasma, *PC* platelet concentrate

The median dose of administered HES was 1000 mL (1000 to 1000 mL). All patients additionally received crystalloid solution with a median dose of 3500 mL (2500 to 3500 mL).

### Renal function

**Fig. 2:**
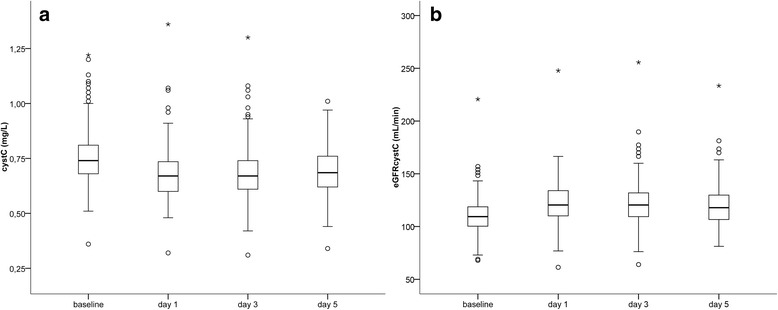
Box-whisker-plots on perioperative renal function. Illustration of the course of peri-operative **a** serum cystatin C concentration (cystC) and **b** corresponding cystC-derived estimated glomerular filtration rate (eGFR_cystC_) in patients undergoing radical prostatectomy (open or robot-assisted). *N* = 179. Wilcox signed rank test: *p* < 0.001 for baseline vs. day 1, day 3, day 5, each

**Fig. 3:**
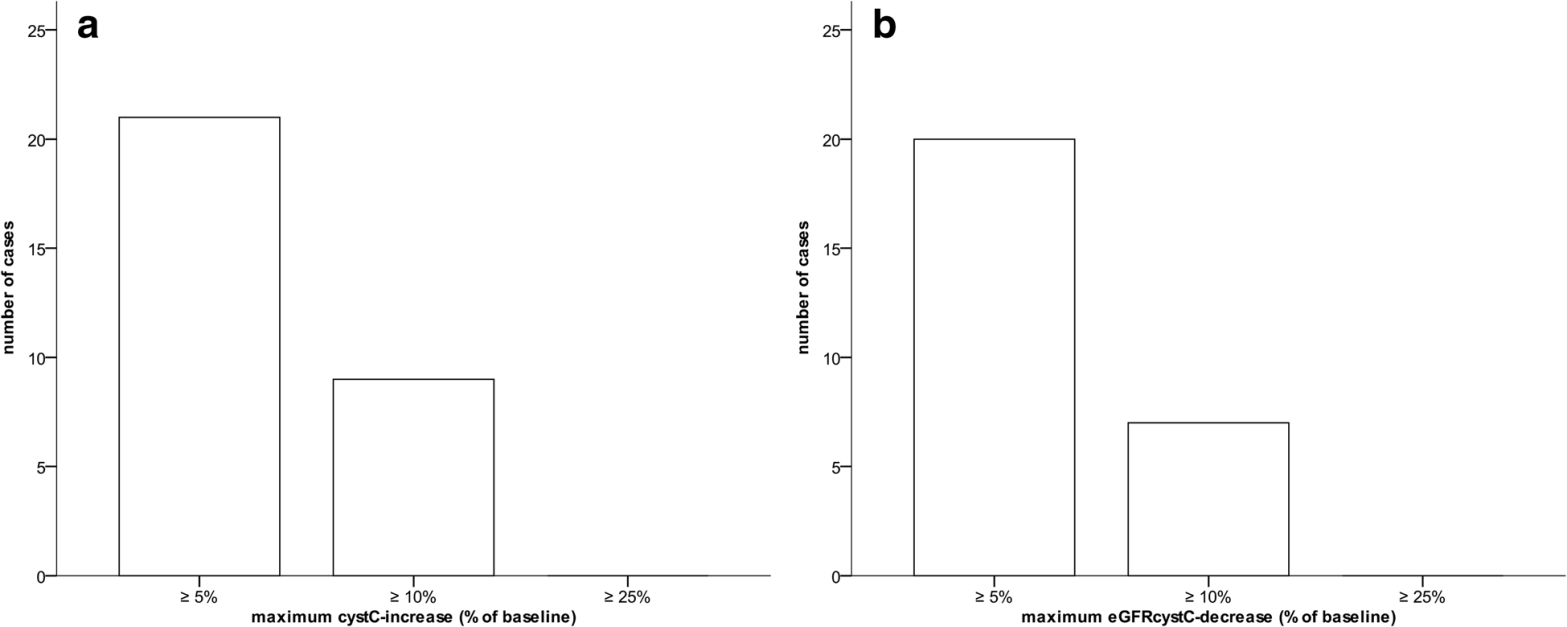
Frequencies of perioperative renal function deterioration. Frequency of maximum perioperative **a** serum Cystatin C (cystC)-increase and **b** cystC-derived estimated glomerular filtration rate (eGFRcystC)-decrease in patients undergoing radical prostatectomy (open or robot-assisted) on postoperative days 1, 3 or 5. *N* = 179

We show data on baseline renal function and renal function on postoperative days 1, 3, and 5 in Table 3 and Figs. 2a, b, 3a, and b. Postoperative cystC values were available for 1, 2, and 3 postoperative days in 12 (6.7 %), 53 (29.6 %), and 114 (63.7 %) patients, respectively.

**Table 3:** Renal function

Parameter, unit	Number of patients with available data, n	Value
Graded baseline eGFR_cystC_, n	179	
≥ 90 mL/min		157 (87.7)
60–89 mL/min		22 (12.3)
30–59 mL/min		0 (0.0)
15–29 mL/min		0 (0.0)
< 15 mL/min		0 (0.0)
Serum cystatin C, mg/L		
Preoperative baseline	179	0.74 (0.68 to 0.81)
Postoperative day 1	171	0.67 (0.60 to 0.74)*
Postoperative day 3	157	0.67 (0.61 to 0.74)*
Postoperative day 5	132	0.69 (0.62 to 0.76)*
Serum creatinine, mg/dL		
Preoperative baseline	179	1.00 (0.80 to 1.10)
Postoperative day 1	178	0.90 (0.80 to 1.10)
Postoperative day 3	163	0.90 (0.80 to 1.00)
Postoperative day 5	136	0.90 (0.90 to 1.00)
eGFR_cystC_, mL/min		
Preoperative baseline	179	109.4 (100.3 to 118.7)
Postoperative day 1	171	120.4 (109.4 to 134.0)*
Postoperative day 3	157	120.4 (109.4 to 132.9)*
Postoperative day 5	132	117.9 (106.6 to 129.8)*
eGFR_crea_, mL/min		
Preoperative baseline	179	83.4 (72.1 to 100.4)
Postoperative day 1	178	88.3 (72.0 to 101.1)
Postoperative day 3	163	88.4 (77.9 to 102.5)
Postoperative day 5	136	88.0 (77.9 to 94.5)

Graded eGFR_cystC_ = number of patients with preoperative baseline eGFR_cystC_ values within one of the listed ranged (no patients has a preoperative baseline eGFR_cystC_-value below 60 mL/min; eGFR_cystC_ = serum cystatin C-derived and eGFR_crea_ = serum creatinine-derived glomerular filtration rate. All values are presented as median (25th to 75th percentile)

^*^ *p* < 0.001 vs. preoperative baseline

The number of patients with increases in cystC and decreases in eGFR_cystC_ of ≥5 % and of ≥10 % on either of the postoperative days 1, 3, or 5, compared with the preoperative baseline values were 21 (11.7 %) and 9 (5.0 %) patients, respectively, and 20 (11.2 %) and 7 (3.9 %) patients, respectively. No patient had a preoperative baseline eGFR_cystC_ value below 60 mL/min. Of note, there was no patient with an increase in cystC or decrease in GFR_cystC_ of ≥25 %.

Compared with the median baseline values there was a statistically significant decrease in median cystC values and a statistically significant increase in median eGFR_cystC_ values on postoperative day 1, 3, and 5 (*p* < 0.001, each).

There was no statistically significant difference between median sCr and eGFR_crea_ values on postoperative days 1, 3, and 5 compared with baseline.

## Discussion

The results of this retrospective analysis indicate that the administration of a median dose of 1000 ml 6 % HES 130/0.4 is not associated with a postoperative deterioration of renal function in terms of a decrease in eGFR_cystC_ in patients with normal to near-normal baseline renal function undergoing radical prostatectomy.

Compared with sCr, CystC is a more accurate and precise estimator of a near-normal eGFR from 60 to 90 mL/min [17, 18]. It further exhibits a higher sensitivity for eGFR-changes [11, 12]. Thus, it may detect postoperative AKI earlier as opposed to sCr [19], i.e. as early as one day postoperatively with peak values on day three [20]. Therefore, a possible increase in cystC due to surgery-related AKI should have likely been detected by cystC-measurements during the observed time span in this study.

There are some, mainly smaller clinical studies using cystC for the investigation of the effect of perioperative 6 % HES 130/0.4 on renal function in individuals without acute systemic disease. In a randomised controlled trial (RCT), Mukhtar and colleagues tested 6 % HES 130/0.4 against human 5 % albumin in 40 living donor liver transplant recipients and found no difference in the perioperative course of cystC as well as creatinine clearance [14]. Harten and colleagues analysed data from 29 patients undergoing emergency abdominal surgery in their pilot RCT to investigate the influence of goal directed haemodynamic therapy on kidney function using 6 % HES 130/0.4 in the intervention group vs. usual care for the control-group, while the latter received unspecified colloids different than HES. In their study, there was no statistically significant difference in cystC or sCr between groups [21]. These findings as well as our own results provide some evidence that there are no harmful effects of HES on the GFR in surgical patients in the absence of critical illness.

These findings are in line with a study analysing kidney biomarkers other than cystC in 40 patients undergoing ORP randomised to receive either 6 % HES 130/0.4 or 0.9 % saline perioperatively [22]. In this study, there was no deleterious effect in the HES compared to the normal saline group with regard to sCr, creatinine clearance, neutrophil gelatinase associated lipocalin, as well as the markers of the renin-angiotensin-aldosterone-pathway [22].

A meta-analysis of 19 RCTs including studies on the influence of various 6 % HES solutions on hospital mortality and AKI or renal replacement therapy in surgical patients revealed no statistically significant differences in outcomes in the patients receiving HES compared to the patients receiving the respective alternative solutions [7].

Our results show, that there is not only no increase in cystC levels from baseline values, but that there is even a decrease in cystC. We did not expect the corresponding eGFR_cystC_ to increase in the context of major surgery, which is associated with blood loss and catecholamine use. To offer an explanation, a recent study with healthy volunteers showed that infusion of fluids, including 6 % HES 130/0.4, even though net cystC serum content rises, leads to a dilution of serum cystC concentration early after the infusion [23]. However, as 6 % HES 130/0.4 has a terminal half-life of 16.1 h independent of the degree of pre-existing renal impairment [24], in theory, postoperatively rather than intraoperatively infused fluids may have caused such an effect on cystC on day 3 or 5 postoperatively. Moreover, it has been shown that an increase in extracellular fluid volume leads to increased GFR above baseline values in patients without renal impairment, partly compensating for the extracellular fluid increase, as long as the patients do not exhibit increased extracellular volume at baseline due to fluid overload caused by pre-existing chronic kidney disease [25]. A relatively high ratio of administered fluid volume and blood loss in our patients may have led to this dilution effect. This may be interpreted as an acute compensatory increase in GFR in patients without kidney disease as well as falsely high postoperative eGFR_cystC_ values as a result of a dilution effect on cystC serum concentration.

Risk factors for postoperative AKI have been identified for general surgical [3] as well as urological [26] patients. While urological surgery per se is frequently associated with AKI, the patient subgroup studied here is at no particularly increased risk.

Previous clinical trials have demonstrated adverse effects of HES on renal function and need for renal replacement therapy in patients with severe sepsis [4–6] which has led to the extension of the contraindications of HES for critically ill patients in general [27,28]. Moreover, concerns have been raised, that the use of HES in patients without severe systemic disease may be unsafe, as well [29], however, convincing evidence concerning this matter is still lacking. The main result of the current study, along with other studies, builds the hypothesis that there may be a differential effect of HES on GFR depending on the study population and the clinical context.

Our study has several limitations. The retrospective nature of the study is a major limitation of our study and might restrict generalisability of the results. In addition, we made the assumption that all bags of fluids and blood products were infused or transfused entirely. In addition, it was assumed that all patients were of Caucasian ethnicity. None of the patients in this study exhibited a perioperative rise in cystC, where the corresponding fall in eGFR_cystC_ would be considered relevant for the diagnosis of AKI according to Risk, Injury, Failure, Loss, and End-stage kidney disease (RIFLE) classification criteria [30]. As a result, further analyses aiming at identifying possible influencing factors on perioperative AKI are inapplicable. This study solely focused on eGFR as a parameter of kidney function, while the novel set of structural biomarkers may be considered useful for future prospective studies [31]. Due to its retrospective design, it was not possible to exclude influencing factors on cystC other than the intraoperative fluid therapy. This study can neither make a statement on extra-renal side effects of HES in the study population (e.g. coagulopathy, oedema, cardiac decompensation or pruritus) nor on potential benefits from HES (e.g. haemodynamics, morbidity or mortality), as it was designed to test the hypothesis that HES does not exert harmful effects on kidney function, hence, no final conclusion on the risk/benefit-ratio of HES in this population can be given.

## Conclusions

In conclusion, our study indicates that the administration of a median dose of 1000 mL 6 % HES 130/0.4 seems not to be associated with a clinically relevant perioperative deterioration of renal function in terms of a decrease in eGFR_cystC_ in patients with normal to near-normal baseline renal function undergoing radical prostatectomy. As HES has continued to be in clinical use, large multicentre randomised controlled trials to confirm this notion seem warranted, in which sensitive markers of kidney function and structure should be used and potential benefits from HES should also be considered.

## Abbreviations

AKI: Acute kidney injury
cystC: Cystatin C
eGFR: Estimated glomerular filtration rate
eGFR_crea_: Serum creatinine-derived estimated glomerular filtration rate
eGFR_cystC_: Serum cystatin C-derived estimated glomerular filtration rate
GFR: Glomerular filtration rate
HES: Hydroxyethyl starch
MDRD-4: Simplified Modification of Diet in Renal Disease
ORP: Open radical prostatectomy
RARP: Robot-assisted radical prostatectomy
RCT: Randomised controlled trial
RIFLE: Risk, Injury, Failure, Loss, and End-stage kidney disease
SAP: Systolic arterial blood pressure
sCr: Serum creatinine
